# 2-(2-Hydroxy­ethyl­amino)-3-phenyl-1-benzofuro[3,2-*d*]pyrimidin-4(3*H*)-one dichloro­methane hemisolvate

**DOI:** 10.1107/S1600536809017814

**Published:** 2009-05-20

**Authors:** Zheng-Hong Zhang, Xiao-Ling Liu, Long-Ju Chen

**Affiliations:** a Department of Neurology, Affiliated Renmin Hospital, Yunyang Medical College, Shiyan 442000, People’s Republic of China; b Department of Human Anatomy, Yunyang Medical College, Shiyan 442000, People’s Republic of China; c Department of Medicinal Chemistry, Yunyang Medical College, Shiyan 442000, People’s Republic of China

## Abstract

In the title compound, C_18_H_15_N_3_O_3_·0.5CH_2_Cl_2_, the fused ring benzofuro[2,3-*d*]pyrimidine system is essentially planar [maximum deviation 0.029 (1) Å]. The planes of the pyrimidinone and phenyl rings are nearly perpendicular [dihedral angle = 87.50 (14)°]. The packing of the mol­ecules in the crystal structure is governed mainly by inter­molecular O—H⋯O and N—H⋯O hydrogen-bonding inter­actions and inter­molecular π–π inter­actions between benzofuro[3,2-*d*]pyrimidine units [the interplanar distances are *ca* 3.4 and 3.5 Å, and the distances between adjacent ring centroids are in the range 3.64 (1)–3.76 (1) Å]. The dichloromethane solvent molecule lies on a special position.

## Related literature

For the preparation and biological activity of benzofuropyrimidine derivatives, see: Moneam *et al.* (2004 ▶); Bodke *et al.* (2003 ▶). For π-π stacking inter­actions, see: Hu *et al.* (2005 ▶, 2006 ▶, 2007 ▶, 2008 ▶); Janiak (2000 ▶). For the structures of other fused pyrimidinone derivatives, see: Hu *et al.* (2005 ▶, 2006 ▶, 2007 ▶, 2008 ▶).
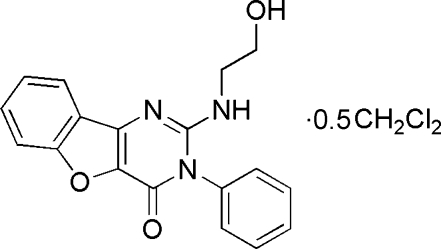


## Experimental

### 

#### Crystal data

C_18_H_15_N_3_O_3_·0.5CH_2_Cl_2_
*M* *_r_* = 363.80
Monoclinic, 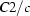

*a* = 26.928 (2) Å
*b* = 7.8931 (7) Å
*c* = 17.3134 (15) Å
β = 110.638 (2)°
*V* = 3443.7 (5) Å^3^
*Z* = 8
Mo *K*α radiation
μ = 0.25 mm^−1^
*T* = 292 K
0.30 × 0.20 × 0.20 mm

#### Data collection

Bruker SMART 4K CCD area-detector diffractometer
Absorption correction: multi-scan (*SADABS*; Sheldrick, 2003 ▶) *T*_min_ = 0.941, *T*_max_ = 0.960
6773 measured reflections
3008 independent reflections
2321 reflections with *I* > 2σ(*I*)
*R*_int_ = 0.025

#### Refinement

*R*[*F* ^2^ > 2σ(*F* ^2^)] = 0.066
*wR*(*F* ^2^) = 0.203
*S* = 1.04
3008 reflections
232 parameters
H-atom parameters constrained
Δρ_max_ = 0.74 e Å^−3^
Δρ_min_ = −0.46 e Å^−3^

### 

Data collection: *SMART* (Bruker, 2001 ▶); cell refinement: *SAINT-Plus* (Bruker, 2001 ▶); data reduction: *SAINT-Plus*; program(s) used to solve structure: *SHELXS97* (Sheldrick, 2008 ▶); program(s) used to refine structure: *SHELXL97* (Sheldrick, 2008 ▶); molecular graphics: *PLATON* (Spek, 2009 ▶); software used to prepare material for publication: *SHELXTL* (Sheldrick, 2008 ▶).

## Supplementary Material

Crystal structure: contains datablocks I, global. DOI: 10.1107/S1600536809017814/at2780sup1.cif

Structure factors: contains datablocks I. DOI: 10.1107/S1600536809017814/at2780Isup2.hkl

Additional supplementary materials:  crystallographic information; 3D view; checkCIF report

## Figures and Tables

**Table 1:** Hydrogen-bond geometry (Å, °)

*D*—H⋯*A*	*D*—H	H⋯*A*	*D*⋯*A*	*D*—H⋯*A*
N3—H3*B*⋯O3^i^	0.86	2.23	2.903 (3)	136
O3—H3*A*⋯O2^ii^	0.82	1.94	2.744 (3)	167

Symmetry codes: (i) 
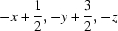
; (ii) 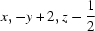.

**Fig. 1.**
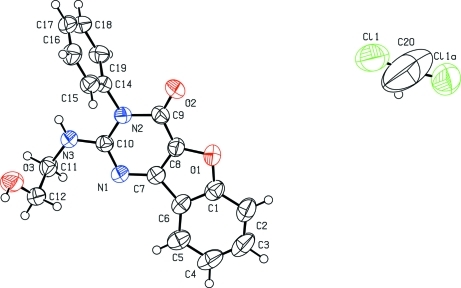
View of the molecular structure of the title compound, showing the atom labelling scheme and with displacement ellipsoids drawn at 50% probability level. [Symmetry code: (a) -x, y, -z +3/2].

**Fig. 2.**
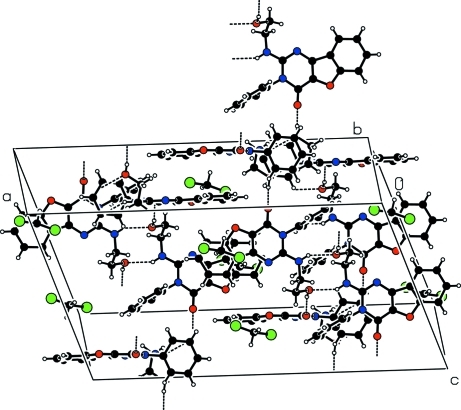
A partial view of the crystal packing of the title compound, showing the hydrogen-bonded stacking interactions (dashed lines).

## supplementary crystallographic information

### Comment

Derivatives of benzofuropyrimidines are of great importance because of their remarkable biological properties, such as the interesting analgesic, antihypertensive, antipyretic, antiviral, and anti-inflammatory activities (Moneam *et al.*, 2004 and Bodke *et al.*, 2003). Some X-ray crystal structures of benzofuro[3,2-*d*]pyrimidinone derivatives have been reported (Hu *et al.*, 2005, 2006, 2007, 2008). The heterocyclic title compound (I) may be used as a new precursor for obtaining bioactive molecules and its structure is presented here (Fig.1).

In the molecule of (I), all ring atoms of benzofuro[2,3-*d*]pyrimidine system are essentially coplanar, with maximum deviations -0.029 (1)Å and 0.027 (2) Å for C7 and N2, respectively. The pyrimidinone ring and the phenyl (C14—C19) ring are nearly perpendicular [dihedral angle = 87.50 (14)]. Intermolecular O—H···O and N—H···O hydrogen-bonding interactions (Table 1) link the molecules, helping to stabilize the crystal structure. Further stability the crystal structure is provided by offset π-π stacking interactions (Janiak, 2000) involving the fused benzofuro[2,3-*d*]pyrimidin system moeties. The interplanar distance are *ca* 3.5 Å, with distances between adjacent ring centroids of 3.6 (1)–3.8 (1)Å [symmetry code relating the adjacent rings: -*x*, 2 - *y*, -*z*].

### Experimental

For background references, see: Hu *et al.* (2008).

### Refinement

All H atoms were located in difference maps and treated as riding atoms with C—H = 0.93 Å, *U*_iso_=1.2*U*_eq_ (C) for C*sp*^2^, C—H = 0.97 Å, *U*_iso_ = 1.2*U*_eq_ (C) for CH_2_, O—H = 0.82 Å, *U*_iso_ = 1.2*U*_eq_ (C) for OH, N—H = 0.86 Å, *U*_iso_ = 1.2*U*_eq_ (N) for NH.

### Crystal data

**Table:**

C_18_H_15_N_3_O_3_·0.5CH_2_Cl_2_	*F*(000) = 1512
*M**_r_* = 363.80	*D*_x_ = 1.403 Mg m^−^^3^
Monoclinic, *C*2/*c*	Mo *K*α radiation, λ = 0.71073 Å
Hall symbol: -C 2yc	Cell parameters from 2289 reflections
*a* = 26.928 (2) Å	θ = 2.5–25.0°
*b* = 7.8931 (7) Å	µ = 0.25 mm^−^^1^
*c* = 17.3134 (15) Å	*T* = 292 K
β = 110.638 (2)°	Needle, colourless
*V* = 3443.7 (5) Å^3^	0.30 × 0.20 × 0.20 mm
*Z* = 8	

### Data collection

**Table:**

Bruker SMART 4K CCD area-detector diffractometer	3008 independent reflections
Radiation source: fine-focus sealed tube	2321 reflections with *I* > 2σ(*I*)
graphite	*R*_int_ = 0.025
φ and ω scans	θ_max_ = 25.0°, θ_min_ = 2.5°
Absorption correction: multi-scan (*SADABS*; Sheldrick, 2003)	*h* = −24→32
*T*_min_ = 0.941, *T*_max_ = 0.960	*k* = −9→9
6773 measured reflections	*l* = −20→20

### Refinement

**Table:**

Refinement on *F*^2^	Secondary atom site location: difference Fourier map
Least-squares matrix: full	Hydrogen site location: inferred from neighbouring sites
*R*[*F*^2^ > 2σ(*F*^2^)] = 0.066	H-atom parameters constrained
*wR*(*F*^2^) = 0.203	*w* = 1/[σ^2^(*F*_o_^2^) + (0.1003*P*)^2^ + 4.9568*P*] where *P* = (*F*_o_^2^ + 2*F*_c_^2^)/3
*S* = 1.04	(Δ/σ)_max_ < 0.001
3008 reflections	Δρ_max_ = 0.74 e Å^−^^3^
232 parameters	Δρ_min_ = −0.46 e Å^−^^3^
0 restraints	Extinction correction: *SHELXL97* (Sheldrick, 2008), Fc^*^=kFc[1+0.001xFc^2^λ^3^/sin(2θ)]^-1/4^
Primary atom site location: structure-invariant direct methods	Extinction coefficient: 0.0023 (4)

### Special details

**Table:**

Geometry. All e.s.d.'s (except the e.s.d. in the dihedral angle between two l.s. planes) are estimated using the full covariance matrix. The cell e.s.d.'s are taken into account individually in the estimation of e.s.d.'s in distances, angles and torsion angles; correlations between e.s.d.'s in cell parameters are only used when they are defined by crystal symmetry. An approximate (isotropic) treatment of cell e.s.d.'s is used for estimating e.s.d.'s involving l.s. planes.
Refinement. Refinement of *F*^2^ against ALL reflections. The weighted *R*-factor *wR* and goodness of fit *S* are based on *F*^2^, conventional *R*-factors *R* are based on *F*, with *F* set to zero for negative *F*^2^. The threshold expression of *F*^2^ > σ(*F*^2^) is used only for calculating *R*-factors(gt) *etc*. and is not relevant to the choice of reflections for refinement. *R*-factors based on *F*^2^ are statistically about twice as large as those based on *F*, and *R*- factors based on ALL data will be even larger.

### Fractional atomic coordinates and isotropic or equivalent isotropic displacement parameters (Å^2^)

**Table:**

	*x*	*y*	*z*	*U*_iso_*/*U*_eq_	Occ. (<1)
C1	0.00474 (12)	0.9366 (4)	0.1209 (2)	0.0562 (8)	
C2	−0.04614 (14)	0.9437 (5)	0.1234 (2)	0.0724 (10)	
H2	−0.0549	1.0163	0.1589	0.087*	
C3	−0.08262 (14)	0.8376 (6)	0.0704 (3)	0.0828 (13)	
H3	−0.1172	0.8374	0.0703	0.099*	
C4	−0.06967 (14)	0.7306 (6)	0.0171 (3)	0.0772 (11)	
H4	−0.0958	0.6618	−0.0185	0.093*	
C5	−0.01884 (12)	0.7234 (4)	0.0155 (2)	0.0626 (9)	
H5	−0.0103	0.6509	−0.0203	0.075*	
C6	0.01922 (11)	0.8287 (4)	0.06958 (18)	0.0502 (7)	
C7	0.07480 (11)	0.8619 (4)	0.08694 (17)	0.0456 (7)	
C8	0.08819 (11)	0.9878 (4)	0.14460 (18)	0.0494 (7)	
C9	0.13833 (11)	1.0657 (4)	0.17189 (17)	0.0488 (7)	
C10	0.15717 (11)	0.8521 (3)	0.08219 (16)	0.0423 (7)	
C11	0.18301 (12)	0.6367 (4)	0.0038 (2)	0.0534 (8)	
H11A	0.1605	0.5601	0.0205	0.064*	
H11B	0.2159	0.5778	0.0106	0.064*	
C12	0.15587 (12)	0.6799 (5)	−0.0862 (2)	0.0617 (9)	
H12A	0.1455	0.5770	−0.1185	0.074*	
H12B	0.1242	0.7464	−0.0936	0.074*	
C14	0.22429 (10)	1.0652 (4)	0.15552 (16)	0.0443 (7)	
C15	0.23170 (12)	1.1918 (4)	0.10617 (18)	0.0525 (8)	
H15	0.2036	1.2268	0.0597	0.063*	
C16	0.28099 (13)	1.2672 (4)	0.1257 (2)	0.0591 (8)	
H16	0.2862	1.3529	0.0925	0.071*	
C17	0.32232 (12)	1.2151 (4)	0.1946 (2)	0.0577 (8)	
H17	0.3555	1.2653	0.2078	0.069*	
C18	0.31458 (12)	1.0894 (4)	0.2437 (2)	0.0603 (9)	
H18	0.3426	1.0553	0.2903	0.072*	
C19	0.26543 (12)	1.0123 (4)	0.22476 (19)	0.0533 (8)	
H19	0.2603	0.9267	0.2581	0.064*	
Cl1	0.04437 (10)	0.5409 (4)	0.71999 (15)	0.1632 (11)	
N1	0.10909 (9)	0.7873 (3)	0.05548 (14)	0.0463 (6)	
N2	0.17227 (9)	0.9896 (3)	0.13565 (14)	0.0439 (6)	
N3	0.19460 (9)	0.7832 (3)	0.05727 (14)	0.0495 (6)	
H3B	0.2259	0.8269	0.0733	0.059*	
O1	0.04639 (8)	1.0374 (3)	0.16786 (13)	0.0602 (6)	
O2	0.15384 (9)	1.1865 (3)	0.21917 (14)	0.0665 (7)	
O3	0.19168 (9)	0.7736 (4)	−0.11261 (14)	0.0768 (8)	
H3A	0.1774	0.7980	−0.1615	0.115*	
C20	0.0000	0.509 (3)	0.7500	0.394 (18)	
H20A	0.0194	0.4328	0.7943	0.473*	0.5
H20B	−0.0194	0.4329	0.7057	0.473*	0.5

### Atomic displacement parameters (Å^2^)

**Table:**

	*U*^11^	*U*^22^	*U*^33^	*U*^12^	*U*^13^	*U*^23^
C1	0.0478 (17)	0.068 (2)	0.0549 (18)	0.0005 (15)	0.0201 (14)	0.0145 (16)
C2	0.053 (2)	0.099 (3)	0.073 (2)	0.011 (2)	0.0313 (18)	0.018 (2)
C3	0.0431 (19)	0.120 (3)	0.087 (3)	−0.001 (2)	0.0251 (19)	0.029 (3)
C4	0.0456 (19)	0.093 (3)	0.084 (3)	−0.0150 (18)	0.0124 (18)	0.017 (2)
C5	0.0516 (18)	0.061 (2)	0.070 (2)	−0.0065 (15)	0.0149 (16)	0.0112 (17)
C6	0.0435 (16)	0.0517 (17)	0.0555 (17)	0.0010 (13)	0.0178 (13)	0.0140 (14)
C7	0.0438 (15)	0.0473 (16)	0.0449 (15)	−0.0002 (12)	0.0148 (12)	0.0062 (13)
C8	0.0437 (16)	0.0604 (18)	0.0476 (16)	0.0026 (13)	0.0203 (13)	0.0019 (14)
C9	0.0524 (17)	0.0546 (18)	0.0395 (15)	0.0007 (14)	0.0164 (13)	−0.0018 (14)
C10	0.0435 (15)	0.0447 (15)	0.0380 (14)	−0.0031 (12)	0.0134 (11)	0.0018 (12)
C11	0.0513 (17)	0.0456 (16)	0.068 (2)	−0.0010 (13)	0.0265 (15)	−0.0059 (15)
C12	0.0446 (17)	0.073 (2)	0.065 (2)	−0.0032 (15)	0.0161 (15)	−0.0223 (17)
C14	0.0429 (15)	0.0483 (16)	0.0417 (15)	−0.0034 (12)	0.0149 (12)	−0.0063 (13)
C15	0.0495 (17)	0.0603 (19)	0.0460 (16)	−0.0003 (14)	0.0149 (13)	0.0063 (14)
C16	0.0593 (19)	0.0572 (19)	0.067 (2)	−0.0058 (15)	0.0294 (16)	0.0038 (16)
C17	0.0450 (17)	0.0612 (19)	0.069 (2)	−0.0117 (14)	0.0225 (15)	−0.0185 (17)
C18	0.0444 (17)	0.073 (2)	0.0546 (18)	−0.0015 (15)	0.0068 (14)	−0.0076 (17)
C19	0.0525 (18)	0.0566 (18)	0.0469 (17)	−0.0055 (14)	0.0126 (14)	0.0013 (14)
Cl1	0.1220 (17)	0.241 (3)	0.1193 (16)	−0.0455 (18)	0.0331 (14)	−0.0050 (18)
N1	0.0406 (13)	0.0489 (14)	0.0493 (13)	−0.0041 (10)	0.0158 (10)	−0.0024 (11)
N2	0.0413 (13)	0.0491 (14)	0.0412 (12)	−0.0043 (10)	0.0142 (10)	−0.0030 (11)
N3	0.0408 (13)	0.0573 (15)	0.0520 (14)	−0.0054 (11)	0.0182 (11)	−0.0109 (12)
O1	0.0528 (13)	0.0745 (15)	0.0603 (13)	0.0037 (11)	0.0286 (11)	−0.0056 (11)
O2	0.0653 (14)	0.0744 (16)	0.0605 (13)	−0.0098 (12)	0.0229 (11)	−0.0266 (12)
O3	0.0628 (15)	0.110 (2)	0.0520 (13)	−0.0030 (14)	0.0131 (11)	0.0062 (13)
C20	0.137 (13)	0.56 (5)	0.44 (4)	0.000	0.038 (18)	0.000

### Geometric parameters (Å, °)

**Table:**

C1—O1	1.381 (4)	C11—H11A	0.9700
C1—C6	1.383 (5)	C11—H11B	0.9700
C1—C2	1.387 (5)	C12—O3	1.413 (4)
C2—C3	1.369 (6)	C12—H12A	0.9700
C2—H2	0.9300	C12—H12B	0.9700
C3—C4	1.383 (6)	C14—C15	1.375 (4)
C3—H3	0.9300	C14—C19	1.379 (4)
C4—C5	1.380 (5)	C14—N2	1.448 (3)
C4—H4	0.9300	C15—C16	1.383 (4)
C5—C6	1.393 (4)	C15—H15	0.9300
C5—H5	0.9300	C16—C17	1.376 (5)
C6—C7	1.443 (4)	C16—H16	0.9300
C7—N1	1.360 (3)	C17—C18	1.370 (5)
C7—C8	1.364 (4)	C17—H17	0.9300
C8—O1	1.379 (3)	C18—C19	1.387 (4)
C8—C9	1.405 (4)	C18—H18	0.9300
C9—O2	1.230 (4)	C19—H19	0.9300
C9—N2	1.412 (4)	Cl1—C20	1.483 (5)
C10—N1	1.315 (3)	N3—H3B	0.8600
C10—N3	1.343 (3)	O3—H3A	0.8200
C10—N2	1.391 (3)	C20—H20A	0.9700
C11—N3	1.445 (4)	C20—H20B	0.9700
C11—C12	1.508 (5)		
			
O1—C1—C6	112.2 (3)	C11—C12—H12A	110.0
O1—C1—C2	124.3 (3)	O3—C12—H12B	110.0
C6—C1—C2	123.4 (3)	C11—C12—H12B	110.0
C3—C2—C1	116.0 (4)	H12A—C12—H12B	108.4
C3—C2—H2	122.0	C15—C14—C19	120.8 (3)
C1—C2—H2	122.0	C15—C14—N2	119.5 (2)
C2—C3—C4	122.1 (3)	C19—C14—N2	119.7 (3)
C2—C3—H3	119.0	C14—C15—C16	119.8 (3)
C4—C3—H3	119.0	C14—C15—H15	120.1
C5—C4—C3	121.6 (4)	C16—C15—H15	120.1
C5—C4—H4	119.2	C17—C16—C15	119.8 (3)
C3—C4—H4	119.2	C17—C16—H16	120.1
C4—C5—C6	117.5 (4)	C15—C16—H16	120.1
C4—C5—H5	121.2	C18—C17—C16	120.1 (3)
C6—C5—H5	121.2	C18—C17—H17	120.0
C1—C6—C5	119.5 (3)	C16—C17—H17	120.0
C1—C6—C7	105.1 (3)	C17—C18—C19	120.8 (3)
C5—C6—C7	135.4 (3)	C17—C18—H18	119.6
N1—C7—C8	124.5 (3)	C19—C18—H18	119.6
N1—C7—C6	129.7 (3)	C14—C19—C18	118.7 (3)
C8—C7—C6	105.8 (3)	C14—C19—H19	120.7
C7—C8—O1	112.8 (3)	C18—C19—H19	120.7
C7—C8—C9	122.7 (3)	C10—N1—C7	114.5 (2)
O1—C8—C9	124.3 (3)	C10—N2—C9	123.0 (2)
O2—C9—C8	128.6 (3)	C10—N2—C14	120.7 (2)
O2—C9—N2	120.3 (3)	C9—N2—C14	116.3 (2)
C8—C9—N2	111.1 (3)	C10—N3—C11	120.8 (2)
N1—C10—N3	119.1 (3)	C10—N3—H3B	119.6
N1—C10—N2	123.9 (2)	C11—N3—H3B	119.6
N3—C10—N2	116.9 (2)	C8—O1—C1	104.0 (2)
N3—C11—C12	113.4 (3)	C12—O3—H3A	109.5
N3—C11—H11A	108.9	Cl1—C20—Cl1^i^	161 (2)
C12—C11—H11A	108.9	Cl1—C20—H20A	96.0
N3—C11—H11B	108.9	Cl1^i^—C20—H20A	96.0
C12—C11—H11B	108.9	Cl1—C20—H20B	96.0
H11A—C11—H11B	107.7	Cl1^i^—C20—H20B	96.0
O3—C12—C11	108.4 (2)	H20A—C20—H20B	103.4
O3—C12—H12A	110.0		
			
O1—C1—C2—C3	178.0 (3)	C16—C17—C18—C19	0.4 (5)
C6—C1—C2—C3	−0.8 (5)	C15—C14—C19—C18	−0.1 (5)
C1—C2—C3—C4	−0.5 (6)	N2—C14—C19—C18	−178.4 (3)
C2—C3—C4—C5	1.0 (6)	C17—C18—C19—C14	−0.2 (5)
C3—C4—C5—C6	−0.2 (5)	N3—C10—N1—C7	177.5 (2)
O1—C1—C6—C5	−177.3 (3)	N2—C10—N1—C7	−2.4 (4)
C2—C1—C6—C5	1.6 (5)	C8—C7—N1—C10	−3.2 (4)
O1—C1—C6—C7	1.3 (3)	C6—C7—N1—C10	177.5 (3)
C2—C1—C6—C7	−179.8 (3)	N1—C10—N2—C9	5.1 (4)
C4—C5—C6—C1	−1.0 (5)	N3—C10—N2—C9	−174.8 (2)
C4—C5—C6—C7	−179.1 (3)	N1—C10—N2—C14	−173.4 (3)
C1—C6—C7—N1	177.8 (3)	N3—C10—N2—C14	6.7 (4)
C5—C6—C7—N1	−3.9 (6)	O2—C9—N2—C10	179.6 (3)
C1—C6—C7—C8	−1.6 (3)	C8—C9—N2—C10	−1.9 (4)
C5—C6—C7—C8	176.7 (3)	O2—C9—N2—C14	−1.9 (4)
N1—C7—C8—O1	−178.1 (2)	C8—C9—N2—C14	176.7 (2)
C6—C7—C8—O1	1.4 (3)	C15—C14—N2—C10	88.1 (3)
N1—C7—C8—C9	6.4 (5)	C19—C14—N2—C10	−93.5 (3)
C6—C7—C8—C9	−174.1 (3)	C15—C14—N2—C9	−90.5 (3)
C7—C8—C9—O2	175.0 (3)	C19—C14—N2—C9	87.9 (3)
O1—C8—C9—O2	0.0 (5)	N1—C10—N3—C11	−1.8 (4)
C7—C8—C9—N2	−3.5 (4)	N2—C10—N3—C11	178.1 (2)
O1—C8—C9—N2	−178.4 (3)	C12—C11—N3—C10	82.1 (3)
N3—C11—C12—O3	66.0 (3)	C7—C8—O1—C1	−0.6 (3)
C19—C14—C15—C16	0.2 (5)	C9—C8—O1—C1	174.8 (3)
N2—C14—C15—C16	178.6 (3)	C6—C1—O1—C8	−0.5 (3)
C14—C15—C16—C17	−0.1 (5)	C2—C1—O1—C8	−179.4 (3)
C15—C16—C17—C18	−0.2 (5)		

Symmetry codes: (i) −*x*, *y*, −*z*+3/2.

### Hydrogen-bond geometry (Å, °)

**Table:**

*D*—H···*A*	*D*—H	H···*A*	*D*···*A*	*D*—H···*A*
N3—H3B···O3^ii^	0.86	2.23	2.903 (3)	136
O3—H3A···O2^iii^	0.82	1.94	2.744 (3)	167

Symmetry codes: (ii) −*x*+1/2, −*y*+3/2, −*z*; (iii) *x*, −*y*+2, *z*−1/2.
